# SVD-aided pseudo principal-component analysis: A new method to speed up and improve determination of the optimum kinetic model from time-resolved data

**DOI:** 10.1063/1.4979854

**Published:** 2017-04-05

**Authors:** Key Young Oang, Cheolhee Yang, Srinivasan Muniyappan, Jeongho Kim, Hyotcherl Ihee

**Affiliations:** 1 Department of Chemistry, KAIST, Daejeon 34141, South Korea; 2 Center for Nanomaterials and Chemical Reactions, Institute for Basic Science (IBS), Daejeon 34141, South Korea; 3 Department of Chemistry, Inha University, Incheon 22212, South Korea

## Abstract

Determination of the optimum kinetic model is an essential prerequisite for characterizing dynamics and mechanism of a reaction. Here, we propose a simple method, termed as singular value decomposition-aided pseudo principal-component analysis (SAPPA), to facilitate determination of the optimum kinetic model from time-resolved data by bypassing any need to examine candidate kinetic models. We demonstrate the wide applicability of SAPPA by examining three different sets of experimental time-resolved data and show that SAPPA can efficiently determine the optimum kinetic model. In addition, the results of SAPPA for both time-resolved X-ray solution scattering (TRXSS) and transient absorption (TA) data of the same protein reveal that global structural changes of protein, which is probed by TRXSS, may occur more slowly than local structural changes around the chromophore, which is probed by TA spectroscopy.

## I. INTRODUCTION

Characterization of molecular structures of transient species formed during chemical and biologically relevant reactions is necessary for understanding their reaction mechanisms and functions. Over the last decade, time-resolved X-ray solution scattering (TRXSS), also known as time-resolved X-ray liquidography (TRXL), based on 3rd- and 4th-generation light sources has been used to investigate molecular structural dynamics of various solution-phase reactions.^1–68^ In our previous TRXSS studies,^60–68^ especially on proteins,^60–65^ we applied singular value decomposition (SVD) analysis and kinetic analysis to determine the optimum kinetic model that best describes the experimental data. As a result of this SVD-aided kinetic analysis, we obtained both time-dependent concentrations of transient intermediate species and time-independent difference X-ray scattering curves, which are directly associated with the structure of the intermediate species. These species-associated difference X-ray scattering curves (SACs) obeying the optimum kinetic model were further examined to reveal molecular structures of the intermediate species by performing structure refinement.^61,62,65–67^ Thus, determining the optimum kinetic model is an essential prerequisite for characterizing the dynamics of a reaction and molecular structures of transient species formed during the reaction.

**FIG. 1.**
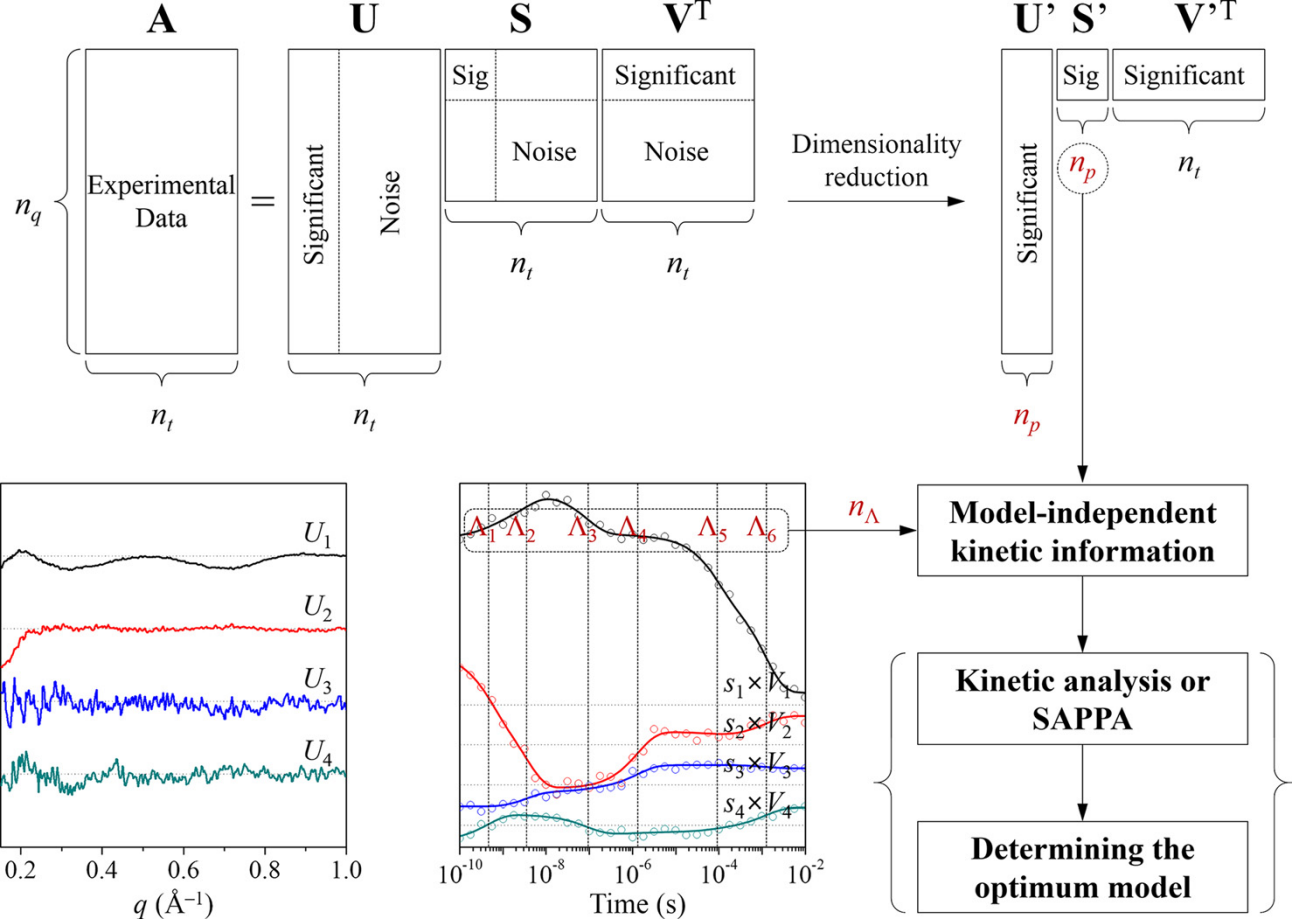
Flowchart of the SVD analysis for TRXSS data. As an example, here we show the case of wild-type sperm whale MbCO where *n_p_* and *n*_Λ_ are 4 and 6, respectively (see the text for details). The model-independent kinetic information obtained from the SVD analysis, such as the number of intermediates (in this case, *n_p_ = *4), the number of relaxation times (in this case, *n*_Λ_ = 6), and their values (in this case, Λ_1_, Λ_2_, Λ_3_, Λ_4_, Λ_5_, and Λ_6_) can be used in the subsequent analysis for determining the optimum kinetic model as illustrated in Figures 2(a) and 2(b).

**FIG. 2.**
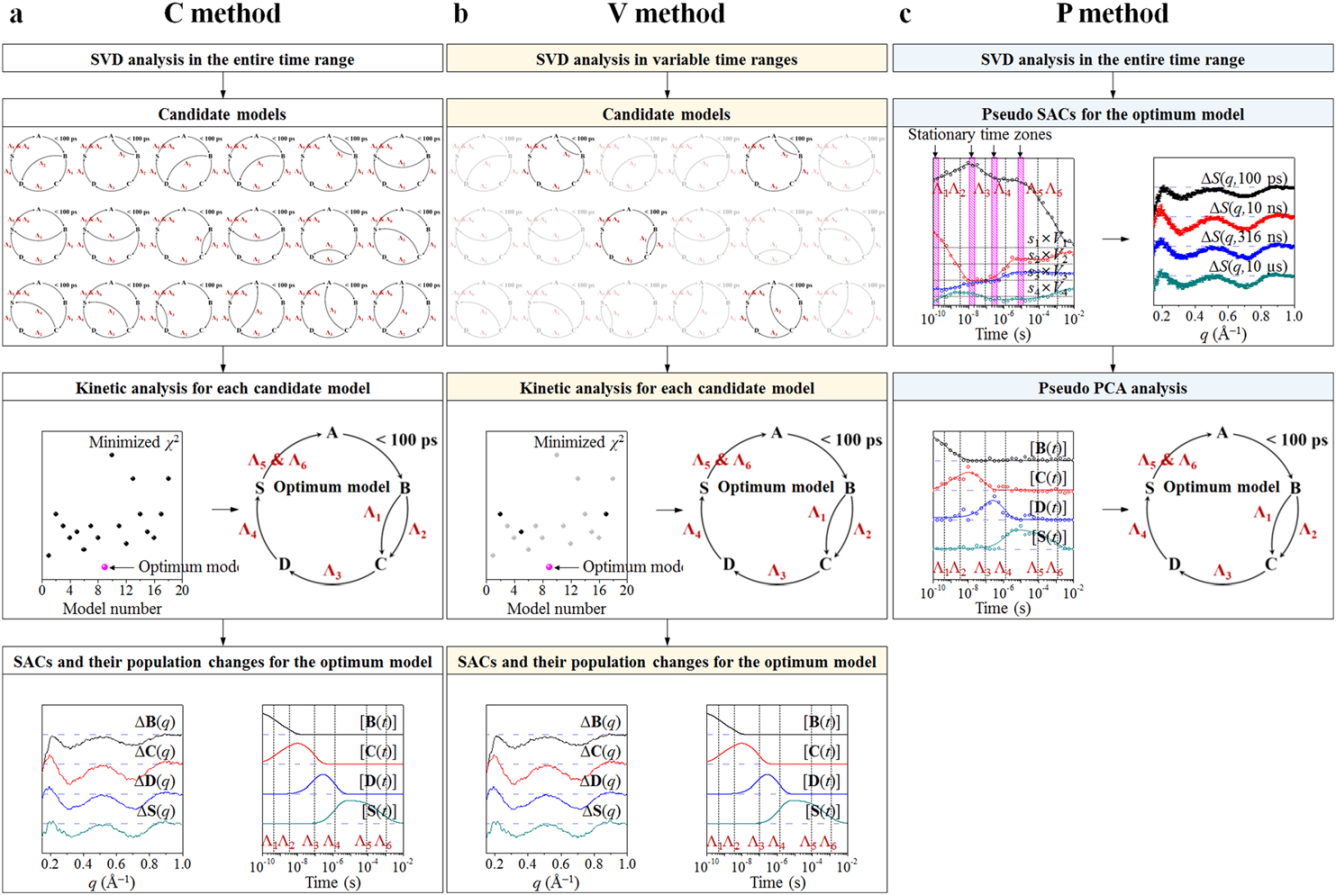
Comparison of three analysis methods for determining the optimum kinetic model from the experimental TRXSS data. For example, here we demonstrate the process of each method for the case of wild-type sperm whale MbCO. (a) In conventional SVD-aided kinetic analysis (termed as C method), we first generate all (in this case, eighteen) possible candidate kinetic models based on the model-independent kinetic information of the SVD analysis in the entire time range (*n_p_*, *n*_Λ_, Λ_1_, Λ_2_, Λ_3_, Λ_4_, Λ_5_, and Λ_6_) as shown in Figure 1. Then, we performed the kinetic analysis for each of the eighteen candidate models. By comparing the minimized *χ*^2^ values of all the candidate models, we finally determined the optimum kinetic model that best fits the experimental data and extracted the species-associated difference X-ray scattering curves (SACs) of the intermediate species for the optimum model. (b) In the second method (termed as the V method), the number of candidate kinetic models can be reduced by additionally performing the SVD analyses in certain reduced time ranges whose upper limits are set to be close to the value of one of the relaxation components obtained from the SVD analysis in the entire time range. By doing so, we had to perform the kinetic analysis only for the four candidate models that are consistent with the SVD analysis in variable reduced time ranges. We note that both the C method and the V method require examination of candidate models against the data. (c) In contrast, SVD-aided pseudo PCA analysis (termed as the P method) can be used to extract the pseudo SACs for the optimum kinetic model directly from the experimental data based on the SVD analysis in the entire time range. Then, the PCA analysis using the pseudo SACs as the time-independent principal components provides the time-dependent population changes of each intermediate from the time-dependent coefficient of the corresponding pseudo SAC. Consequently, the optimum kinetic model can be determined without the need of any candidate kinetic model.

As illustrated in Figure 1, SVD analysis provides model-independent kinetic information, for example, the number of structurally distinct intermediates (*n_p_*) and their associated relaxation times (Λ_*i*_, where *i* = 1,…, *n*_Λ_). Subsequently, kinetic analysis determines the optimum kinetic model among all possible candidate kinetic models that are considered based on the information obtained from the SVD analysis (termed as the C method in Figure 2(a)). For example, in our recent report on direct observation of the bond formation in a gold trimer complex,^66^ SVD analysis on the TRXSS data showed that there exist “three” intermediate states and “three” relaxation times, allowing us to consider only a simple sequential kinetic model in the kinetic analysis. In contrast, in our TRXSS study on wild-type sperm whale myoglobin (Mb),^63^ SVD analysis revealed that there exist “four” intermediates and “six” relaxation times. The fifth and sixth relaxation times correspond to nonexponential recovery of the ground-state Mb liganded with CO molecules from the last (fourth) intermediate, that is, bimolecular nongeminate CO recombination.^69,70^ Because the number of relaxation times is larger than that of intermediates, the optimum kinetic model must contain parallel (that is, biphasic) and/or bypass pathway(s) and thus we considered a total of eighteen candidate kinetic models.

As can be seen in these examples, when the number of relaxation times obtained from the SVD analysis exceeds that of intermediates, the number of candidate kinetic models to be considered in the kinetic analysis increases significantly, making the analysis complicated. Consequently, it is desirable to reduce the number of candidate kinetic models for fast and accurate determination of the optimum kinetic model. In the case of wild-type sperm whale MbCO discussed above,^63^ we devised a new method whereby the SVD analysis was performed in variable time ranges (termed as the V method in Figure 2(b)). By doing so, we identified the number of intermediates involved in specific time ranges of interest and used this additional information as a constraint to reduce the number of candidate kinetic models to be considered in the kinetic analysis. As a result, the number of candidate kinetic models was significantly reduced from eighteen to four.

In this work, we introduce a new method of extracting model-dependent kinetic information directly from the experimental data without considering any candidate kinetic model. In this method termed as the P method in Figure 2(c), from the SVD analysis in the entire time range, we identify stationary time zones where the amplitude of left singular vectors (lSVs) remains constant and define the experimental TRXSS data in such time ranges as pseudo SACs of reaction intermediates. Then, the principal-component analysis of the pseudo SACs provides time-dependent relative population of each intermediate species. In this way, we can determine the optimum kinetic model easily and accurately without considering any candidate kinetic model. We demonstrate the applicability of this SVD-aided pseudo principal-component analysis (SAPPA) by examining experimental TRXSS data for wild-type sperm whale MbCO^63^ and wild-type homodimeric hemoglobin liganded with CO molecules (HbI(CO)_2_).^62^ In addition, we show that the new method can be extended to transient absorption (TA) data on HbI(CO)_2_.

## II. METHODS

### A. Singular value decomposition analysis

In order to determine the optimum kinetic model for a reaction, we need to examine how many distinct transient species are involved in the reaction and how fast the population of each species changes. For this purpose, we first apply the singular value decomposition (SVD) analysis^60–68,71^ to experimental TRXSS data as illustrated in Figure 1. From the experimental scattering curves measured at various time delays, we can build an *n_q_* × *n_t_* matrix **A**, where *n_q_* is the number of *q* points in the scattering curve at a given time-delay point and *n_t_* is the number of time-delay points. The matrix **A** can be decomposed while satisfying the relationship of **A** = **USV**^T^, where **U** is an *n_q_* × *n_t_* matrix whose columns are called left singular vectors (lSVs) (i.e., time-independent *q* spectra) of **A**, **V** is an *n_t_* × *n_t_* matrix whose columns are called right singular vectors (rSVs) (i.e., amplitude changes of **U** as time evolves) of **A**, and **S** is an *n_t_* × *n_t_* diagonal matrix whose diagonal elements are called singular values of **A** and can possess only non-negative values. The matrices **U** and **V** have the properties of **U**^T^**U** = **I**_*nt*_ and **V**^T^**V** = **I**_*nt*_, respectively, where **I**_*nt*_ is the identity matrix. Since the diagonal elements (i.e., singular values) of **S**, which represent the weight of left singular vectors in **U**, are ordered so that s_1_ ≥ s_2_ ≥ ⋯ ≥ s_n_ ≥ 0, lSVs and rSVs on more left are supposed to have larger contribution to the constructed experimental data. In this manner, we can extract the time-independent scattering intensity components from the lSVs and the time evolution of their amplitudes from the rSVs. The former, when combined together, can give the information on the scattering curves of distinct transient species, while the latter contains the information on the population dynamics of the transient species. Thus, the SVD analysis provides a model-independent estimation of the number of structurally distinguishable species and the population dynamics of each species.

### B. SVD-aided kinetic analysis: C method

Using the principal singular vectors with significant singular values obtained from the SVD analysis of the experimental data, we typically perform kinetic analysis (termed as the C method in Figure 2(a)) to determine the optimum kinetic model. Dimensionality-reduced matrices, **U**′, **S**′, and **V**′, which can be generated by removing non-significant singular components from **U**, **S**, and **V**, respectively, are illustrated in Figure 1. In other words, **U**′ is an *n_q_* × *n_p_* matrix containing only the first *n_p_* left singular vectors of **U**, **S**′ is an *n_p_* × *n_p_* diagonal matrix containing the first *n_p_* singular values of **S**, and **V**′ is an *n_t_* × *n_p_* matrix containing the first *n_p_* right singular vectors of **V**. Here, we define a matrix **C**, of which the columns represent time-dependent concentrations of transiently formed intermediate species and can be described by a candidate kinetic model that can be generated on the basis of the SVD analysis. Then, the matrix **C** can be related to **V**′ by using a parameter matrix **P** that satisfies **V**′ = **CP**. In our analysis, **C** is an *n_t_* × *n_p_* matrix containing the time-dependent concentrations of *n_p_* intermediates involved in a reaction of interest, and **P** is an *n_p_* × *n_p_* matrix containing coefficients for the time-dependent concentrations so that the linear combination of concentrations of the *n_p_* intermediates can form the *n_p_* right singular vectors in **V**′. Once **C** is expressed using a set of variable kinetic parameters based on a candidate kinetic model, **P** and **C** can be optimized by minimizing the discrepancy between **V**′ (from the experiment) and **CP** (from the kinetic theory). We perform this optimization for each of the candidate kinetic models and compare the minimized discrepancies of all the kinetic models to determine the optimum kinetic model that best fits the experimental data.

However, standard deviations for **V**′ are not available from the experimental data and thus we instead use the following method to optimize **P** and **C**. Since **V**′ = **CP**, the following relationships hold:

$$A\prime =U\prime S\prime V^{\prime T}=U\prime S\prime {\left(CP\right)}^{T}=U\prime S\prime \left(P^{T}C^{T}\right)=\left(U\prime S\prime P^{T}\right)C^{T}=EC^{T},$$ (1)

where **A**′ is an *n_q_* × *n_t_* matrix that contains theoretical difference scattering curves, ΔS_fit_ (*q_i_*, *t_j_*), at given *q* and *t* values. Theoretical difference scattering curves calculated by using Eq. (1) are compared with the experimental difference scattering curves, and the matrices **P** and **C** are optimized by minimizing the discrepancy (quantified by chi-square, *χ*^2^) between the theoretical and experimental difference scattering curves using the Minuit^72^ package

$$\chi^{2}={\sum_{i=1}^{n_{q}}\sum_{j=1}^{n_{t}}\left(\frac{\Delta S_{\text{exp}\;}(q_{i},t_{j})-\Delta S_{fit}(q_{i},t_{j})}{\sigma_{ij}}\right)}^{2},$$ (2)

where Δ*S_exp_* (*q*, *t*) and Δ*S_fit_* (*q*, *t*) are the experimental and theoretical difference scattering intensities at a given point of (*q_i_*, *t_j_*), respectively, and *σ_ij_* is the experimental standard deviation at (*q_i_*, *t_j_*). As written in Eq. (1), we can define a matrix **E** as **E** = **U′S′P**^T^, that is, a linear combination of the *n_p_* left singular vectors in **U**′ weighted by their singular values in **S**′ with their ratios determined by **P**. Then, the matrix **E**, an *n_q_* × *n_p_* matrix, contains the *n_p_* difference scattering curves directly associated with the *n_p_* intermediate species involved in a reaction of interest. Therefore, by optimizing the matrices **P** and **C** for the optimum kinetic model, we obtain both the time-dependent concentrations (the columns of the optimized **C** for the optimum kinetic model) and the time-independent species-associated difference X-ray scattering curves (SACs) of the intermediate species (the columns of the optimized **E** for the optimum kinetic model).

### C. SVD-aided kinetic analysis with SVD analysis in variable time ranges: V method

The C method described in Section II B becomes complicated and time-consuming when the number of candidate kinetic models increases. Therefore, it is desirable to reduce the number of candidate kinetic models to be examined. Such a goal can be achieved by performing multiple SVD analyses in variable reduced time ranges instead of a single SVD analysis in the entire time range (termed as the V method in Figure 2(b)). An SVD analysis in a reduced time range gives the information on the number of intermediates in that specific time range. This additional information provides a constraint to exclude kinetic models that are not consistent with the SVD analyses in variable reduced time ranges, thus simplifying the determination of the optimum kinetic model. For example, suppose that we identified *n_p_* distinct intermediates and *n_p_* + 1 relaxation times (Λ_*i*_, where *i* = 1,…, *n_p_* + 1) from the SVD analysis in the entire time range of a photoreaction of interest. Assuming that the last relaxation component represents the recovery of the ground state from the last intermediate, the earlier *n_p_* relaxation times must account for the transitions among the *n_p_* intermediates. Since the minimum number of relaxation components required for transitions among *n_p_* intermediates is *n_p_* – 1, one of the relaxation times must be associated with either a parallel (that is, biphasic) pathway or a bypass pathway (to a non-adjacent intermediate). To identify which relaxation component is associated with such a pathway, we can additionally perform the SVD analysis in certain reduced time ranges whose upper limits are set to be close to one of the relaxation times obtained from the SVD analysis in the entire time range. If it turns out that there exist two distinct intermediates in the time range up to around Λ_2_, these two intermediates must be responsible for the first two relaxation times (Λ_1_ and Λ_2_), suggesting the existence of a biphasic or a bypass pathway involving the two intermediates. Consequently, in the subsequent kinetic analysis to determine the optimum kinetic model, we need to consider only the candidate kinetic models consistent with the SVD analyses in variable reduced time ranges, which are a subset of those consistent with the SVD analysis in the entire time range.

For example, in a previous TRXSS study on MbCO,^63^ two relaxation times (460 ps and 3.6 ns) were identified for the first two intermediates (termed **B** and **C**). In general, a transition between two intermediates would exhibit only a single exponential dynamics irrespective of how many relaxation times are assigned for the transition in a kinetic model (see Figure S3 in the supplementary material). However, in the case of MbCO, the first intermediate (**B**) was found to have two conformational substates (termed **B_1_** and **B_2_**) due to the variation of interaction between CO ligand and distal histidine in the primary docking site. Since **B_1_** and **B_2_** have conformations that are only subtly different from each other, their TRXSS patterns are indistinguishable from each other. Despite the structural similarity of the two conformational substates, **B_1_** and **B_2_** transform to **C** with different rate constants. As a result, the transition from **B** to **C** exhibits biphasic dynamics characterized by two relaxation times (see Figure S4 in the supplementary material).

### D. SVD-aided pseudo principal-component analysis: P method

The V method outlined in Section II C still requires that each candidate kinetic model has to be tested against the experimental data and the one that gives the best agreement is chosen as the optimal kinetic model. Here, we introduce a new method, SVD-aided pseudo principal-component analysis (SAPPA), which speeds up and improves determination of the optimum kinetic model from time-resolved data by circumventing such consideration of candidate kinetic models. In principle, the principal lSVs and their time-dependent amplitude changes (that is, principal rSVs) obtained from the SVD analysis provide a basis for the time-independent SACs of reaction intermediates and the time-dependent population changes of those intermediates, respectively. The relationship (**V**′ = **CP**) between the model-independent information (**V**′ in the C method and V method) and the model-dependent information (**C** in the C method and V method) mediated by the matrix **P** in the C method and V method indicates that, if there exist stationary time zones where the amplitudes of all the principal lSVs remain constant, the amplitudes of all the time-independent SACs of the intermediates should also remain constant in each of those time zones. Especially, when the number of such time zones matches the number of intermediates (*n_p_*) identified by the SVD analysis in the entire time range, the experimental time-resolved data in each of the stationary time zones can be directly regarded as the pseudo SAC of each intermediate species (see P method in Figure 2(c)). In other words, without the need of determining the optimum kinetic model, we can easily obtain the optimized matrix **E** whose columns are the SACs for the optimum kinetic model determined in the C method and V method by (i) determining stationary time zones based on the rSVs obtained from the SVD analysis in the entire time range and (ii) taking experimental time-resolved data at the selected stationary time zones as pseudo SACs. Specifically, to systematically determine the stationary time zones, we inspect the sum of the absolute values of the first derivatives of the principal rSVs weighted by singular values with respect to log_10_(time) and take its local minima as stationary time zones as shown in Figure S1 in the supplementary material. Since the original principal rSVs contain noise, the curves fitted to principal rSVs can be used for the calculation of derivatives and the summation of their absolute values. Then, time-resolved data at the selected stationary time zones (that is, pseudo SACs) are used as the columns of the matrix **E** in Eq. (1) and **C** can be obtained by fitting the experimental data at all time delays by linear combinations of the pseudo SACs. In the fitting, the coefficients of the pseudo SACs are determined by minimizing the *χ*^2^ value defined in Eq. (2), and these coefficients correspond to the time-dependent relative populations of the transient intermediate species. Then, by fitting these time-dependent populations with the relaxation times obtained from the SVD analysis in the entire time range, we can easily assign the relaxation components to specific transitions among the intermediates. In this way, we can determine the optimum kinetic model without considering any candidate kinetic model, in contrast to the C method or V method.

## III. RESULTS AND DISCUSSION

### A. SAPPA for TRXSS data of MbCO

**FIG. 3.**
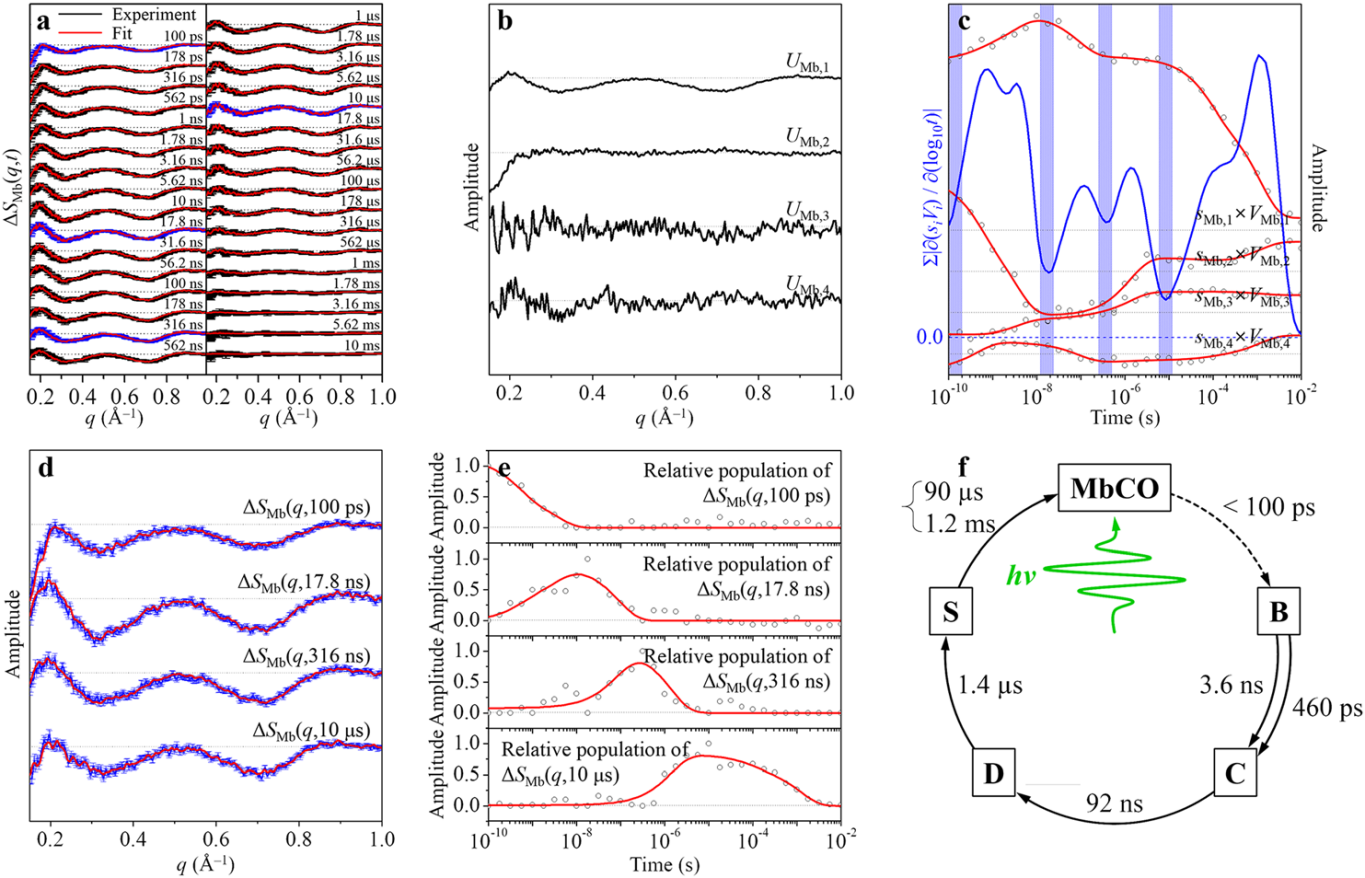
Example of the P method (SVD-aided pseudo PCA analysis) applied to TRXSS data. (a) Time-resolved difference X-ray solution scattering curves, Δ*S*_Mb_(*q*,*t*), measured for a solution sample of wild-type sperm whale MbCO. The time delay after photoexcitation is indicated above each curve. Experimental curves (black curves) are compared with fit curves (red curves) that were generated from the P method. (b) Four principal lSVs. (c) Four principal rSVs weighted by singular values (black circles). These time-dependent singular components were fit (red curves) by six exponentials sharing common relaxation times, yielding the relaxation times of 460 ± 160 ps, 3.6 ± 0.7 ns, 92 ± 25 ns, 1.4 ± 0.2 *μ*s, 90 ± 20 *μ*s, and 1.2 ± 0.2 ms. Sum of the absolute values of the first derivatives of the principal rSVs weighted by singular values with respect to log_10_(time) (blue curve) was used as the selection criteria of the stationary time zones. Within each of the four time zones shaded in blue color, all the weighted principal rSVs remain stationary in their amplitudes, indicating that the experimental curves should not change in these time zones. We took the experimental curves within each of these time zones as pseudo SACs for the four intermediates of MbCO as shown in (d). (d) Pseudo SACs (blue curves) for the four intermediates corresponding to the experimental curves at four selected time delays, 100 ps, 17.8 ns, 316 ns, and 10 *μ*s. These pseudo SACs are compared with the formal SACs (red curves) corresponding to **B**, **C**, **D**, and **S** intermediates of MbCO extracted from the SVD-aided kinetic analysis (C method or V method). (e) Time-dependent relative populations (black circles) of the corresponding pseudo SACs. These populations were fit (red curves) by the relaxation times (460 ps, 3.6 ns, 92 ns, 1.4 *μ*s, 90 *μ*s, and 1.2 ms) obtained in (c). (f) The optimum kinetic model that best describes the structural dynamics of MbCO. This optimum model determined by the P method is identical to the one determined by the C method or the V method.

Time-resolved difference X-ray solution scattering curves, Δ*S*_Mb_(*q*,*t*), measured following photoexcitation of a wild-type sperm whale MbCO solution,^63^ are shown in Figure 3(a). From SVD of the experimental data in the *q* range of 0.15–1.0 Å^−1^ and the entire time range (100 ps–10 ms), we identified four principal singular components, which correspond to four structurally distinct intermediates, and six relaxation times (in this case, four unimolecular time constants and one bimolecular time constant approximated by a combination of two latest unimolecular time constants^69,70^) as shown in Figures 3(b) and 3(c). If the C method is applied, a total of 18 candidate kinetic models have to be considered (see Figure 2(a)). In contrast, if the V method is applied, the number of candidate kinetic models is reduced down to four (see Figure 2(b)), as was done in our previous work on MbCO.^63^ In this work, instead of considering any candidate kinetic model, we applied the P method to determine the optimum kinetic model. Since the number of principal singular vectors was determined to be four from the SVD analysis, we selected four stationary time zones (see Figure 3(c)). Specifically, based on the time-dependent rSVs (black circles (experimental) and red curves (fit) in Figure 3(c)) and the sum of the absolute values of the first derivatives of the principal rSVs weighted by singular values with respect to log_10_(time) (blue curve in Figure 3(c)), we selected 100 ps, 17.8 ns, 316 ns, and 10 *μ*s as stationary time zones and, accordingly, the experimental curves measured at 100 ps, 17.8 ns, 316 ns, and 10 *μ*s as the pseudo SACs corresponding to the four intermediates, as shown in Figure 3(d). We fitted the experimental curves at all time delays by linear combinations of the pseudo SACs and determined the time-dependent relative population of each intermediate from the coefficient of the corresponding pseudo SAC as shown in Figure 3(e). Then, we fitted the time-dependent relative population of each intermediate by the relaxation components obtained from the SVD analysis and assigned each relaxation component to a specific transition.

As shown in Figure 3(e), the relative populations of the first (100 ps), the second (17.8 ns), the third (316 ns), and the fourth (10 *μ*s) pseudo SACs were fit by multiple exponentials. The population of the first pseudo SAC, Δ*S*_Mb_(*q*,100 ps), decays biphasically with time constants of 460 ps and 3.6 ns. Accordingly, the population of the second pseudo SAC, Δ*S*_Mb_(*q*,17.8 ns), rises biphasically with time constants of 460 ps and 3.6 ns and decays with a time constant of 92 ns. Subsequently, the population of the third pseudo SAC, Δ*S*_Mb_(*q*,316 ns), rises with a time constant of 92 ns and decays with a time constant of 1.4 *μ*s. Then, the population of the fourth pseudo SAC, Δ*S*_Mb_(*q*,10 *μ*s), rises with a time constant of 1.4 *μ*s and decays nonexponentially, which can be approximated by two dummy time constants of 90 *μ*s and 1.2 ms.^69,70^ These time-dependent relative populations of pseudo SACs allow us to deduce the optimum kinetic model, which involves (1) biphasic transition from the first intermediate to the second one due to the existence of two conformational substates of the first intermediate and (2) bimolecular nongeminate CO recombination of the fourth intermediate as shown in Figure 3(f). In fact, the kinetic model shown in Figure 3(f) is identical to the optimum kinetic model determined by the V method in our previous work.^63^

### B. SAPPA for TRXSS data of HbI(CO)_2_

**FIG. 4.**
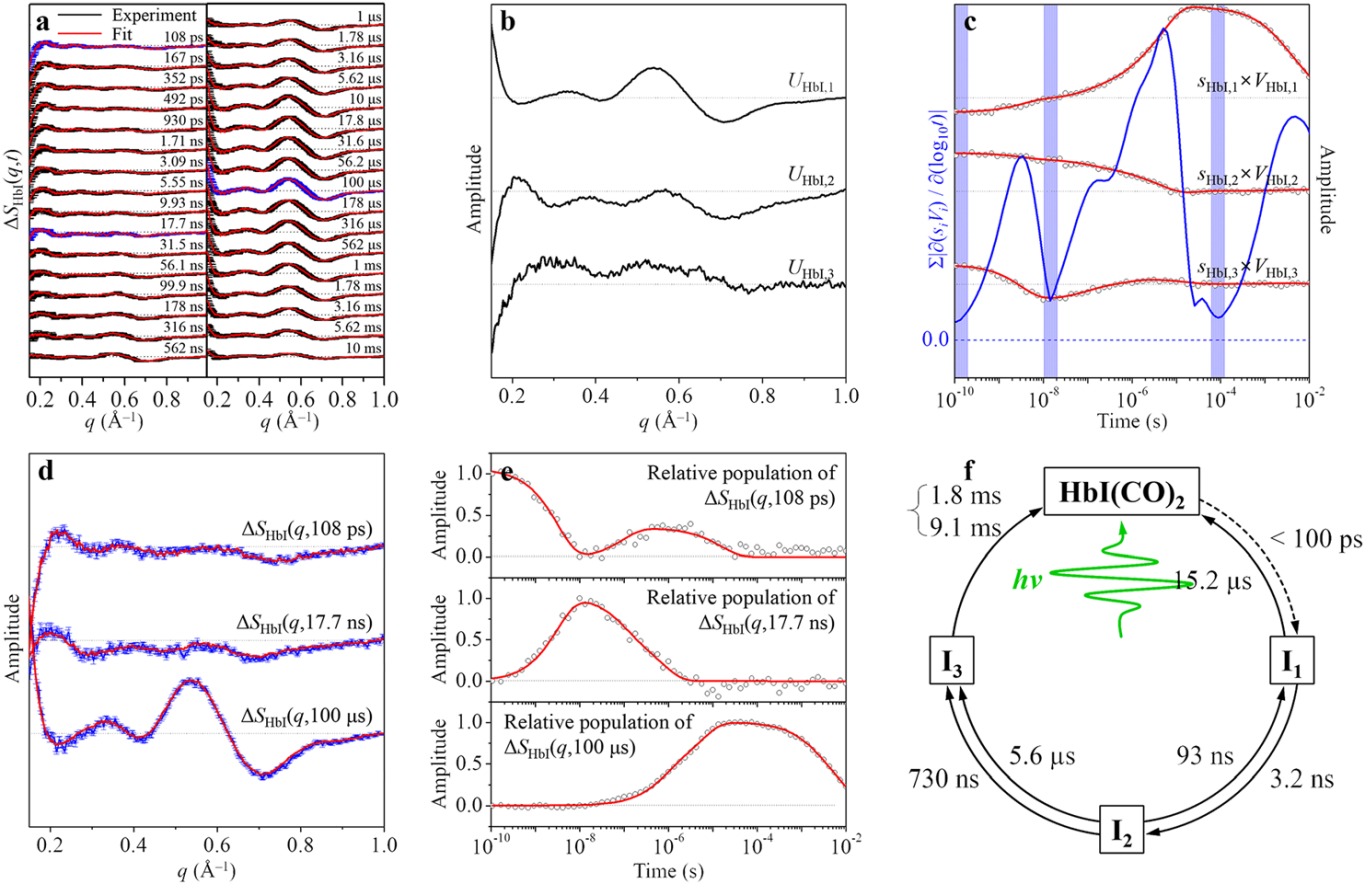
Example of the P method applied to TRXSS data of wild-type HbI(CO)_2_. (a) Time-resolved difference X-ray solution scattering curves, Δ*S*_HbI_(*q*,*t*), measured for a solution sample of wild-type HbI(CO)_2_. The time delay after photoexcitation is indicated above each curve. Experimental curves (black curves) are compared with fit curves (red curves) that were obtained from the P method. (b) Three principal time-independent lSVs. (c) Three principal time-dependent rSVs weighted by singular values (black circles). These time-dependent singular components were fit (red curves) by seven exponentials sharing common relaxation times, yielding the relaxation times of 3.2 ± 0.2 ns, 93 ± 20 ns, 730 ± 120 ns, 5.6 ± 0.8 *μ*s, 15.2 ± 8 *μ*s, 1.8 ± 0.3 ms, and 9.1 ± 0.9 ms. Sum of the absolute values of the first derivatives of the principal rSVs weighted by singular values with respect to log_10_(time) (blue curve) was used as the selection criteria of the stationary time zones. Within each of the three time zones shaded in blue color, all the weighted principal rSVs remain stationary in their amplitudes, and thus the experimental curves should not change in these time zones. We took the experimental curves within each of these time zones as pseudo SACs for the three intermediates of HbI(CO)_2_ as shown in (d). (d) Pseudo SACs (blue curves) for the three intermediates of HbI(CO)_2_ corresponding to the experimental curves at three selected time delays, 108 ps, 17.7 ns, and 100 *μ*s. These pseudo SACs are compared with the formal SACs (red curves) corresponding to I_1_, I_2_, and I_3_ intermediates of HbI(CO)_2_ extracted from the C method or the V method. For clarity, we scaled the formal SAC of I_3_ species to consider the portion of geminate recombination obtained from C or V methods. (e) Time-dependent relative populations (black circles) of the corresponding pseudo SACs. These populations were fit (red curves) by the relaxation times (3.2 ns, 93 ns, 730 ns, 5.6 *μ*s, 15.2 *μ*s, 1.8 ms, and 9.1 ms) obtained in (c). (f) The optimum kinetic model that best describes the structural dynamics of HbI(CO)_2_. This optimum model determined by the P method is identical to the one determined by the C method or V method.

Time-resolved difference X-ray solution scattering curves, Δ*S*_HbI_(*q*,*t*), measured following photoexcitation of a wild-type HbI solution^62^ are shown in Figure 4(a). The measured data were analyzed by applying the P method to determine the optimum kinetic model. From SVD of the experimental data in the *q* range of 0.15–1.0 Å^−1^ and the entire time range (100 ps–56.2 ms), we identified three principal singular components (that is, three structurally distinct intermediates) and seven relaxation times (in this case, five unimolecular time constants and one bimolecular time constant approximated by a combination of two latest unimolecular time constants^69,70^) as shown in Figures 4(b) and 4(c). Since the number of principal singular vectors is three, we selected three stationary time zones (see Figure 4(c)), which are 108 ps, 17.7 ns, and 100 *μ*s based on the time-dependent rSVs (black circles (experimental) and red curves (fit) in Figure 4(c)) and the sum of the absolute values of the first derivatives of the principal rSVs weighted by singular values with respect to log_10_(time) (blue curve in Figure 4(c)). In other words, we selected the experimental curves measured at 108 ps, 17.7 ns, and 100 *μ*s as the pseudo SACs corresponding to the three intermediates, as shown in Figure 4(d). We fitted the experimental curves at all time delays by linear combinations of the pseudo SACs and determined the time-dependent relative population of each intermediate from the coefficient of the corresponding pseudo SAC as shown in Figure 4(e). Then, we fitted the time-dependent relative population of each intermediate by the relaxation components obtained from the SVD analysis and assigned each relaxation component to a specific transition.

As shown in Figure 4(e), the relative populations of the first (108 ps), the second (17.7 ns), and the third (100 *μ*s) pseudo SACs were fit by multiple exponentials. The population of the first pseudo SAC, Δ*S*_HbI_(*q*,108 ps), decays with a time constant of 3.2 ns, rises with a time constant of 93 ns, and decays again with a time constant of 15.2 *μ*s. The population of the second pseudo SAC, Δ*S*_HbI_(*q*,17.7 ns), rises with a time constant of 3.2 ns and decays with time constants of 93 ns, 730 ns, and 5.6 *μ*s. Then, the population of the third pseudo SAC, Δ*S*_HbI_(*q*,100 *μ*s), rises biphasically with time constants of 730 ns and 5.6 *μ*s and decays nonexponentially, which can be approximated by two dummy time constants of 1.8 ms and 9.1 ms.^69,70^ These time-dependent relative populations of pseudo SACs allow us to determine the optimum kinetic model, which involves (1) biphasic transition from the second intermediate to the third one due to the existence of two conformational substates of the second intermediate, (2) geminate CO recombination of the second intermediate, and (3) bimolecular nongeminate CO recombination of the third intermediate as shown in Figure 4(f). In fact, the kinetic model shown in Figure 4(f) is identical to the optimum kinetic model determined by the C method or V method.^62^ We also tested how the selection of stationary time zones affects the analysis result by examining time-dependent relative populations of the three pseudo SACs extracted from seven different combinations of stationary time zones as tabulated in Figure S2 in the supplementary material. As the stationary time zones for the first, the second, and the third pseudo SACs deviate from 108 ps, 17.7 ns, and 100 *μ*s, respectively, the fit to the experimental data becomes worse with increased *χ*^2^ value as shown in Figure S2d in the supplementary material. As long as the first, the second, and the third time zones are selected in the ranges of 108–492 ps, 10 ns–42.2 ns, and 17.8–422 *μ*s, respectively, we found that the analysis result does not change significantly, indicating that the selection of proper stationary time zones should not be difficult (see Figure S1 in the supplementary material).

### C. SAPPA for transient absorption data of HbI(CO)_2_

**FIG. 5.**
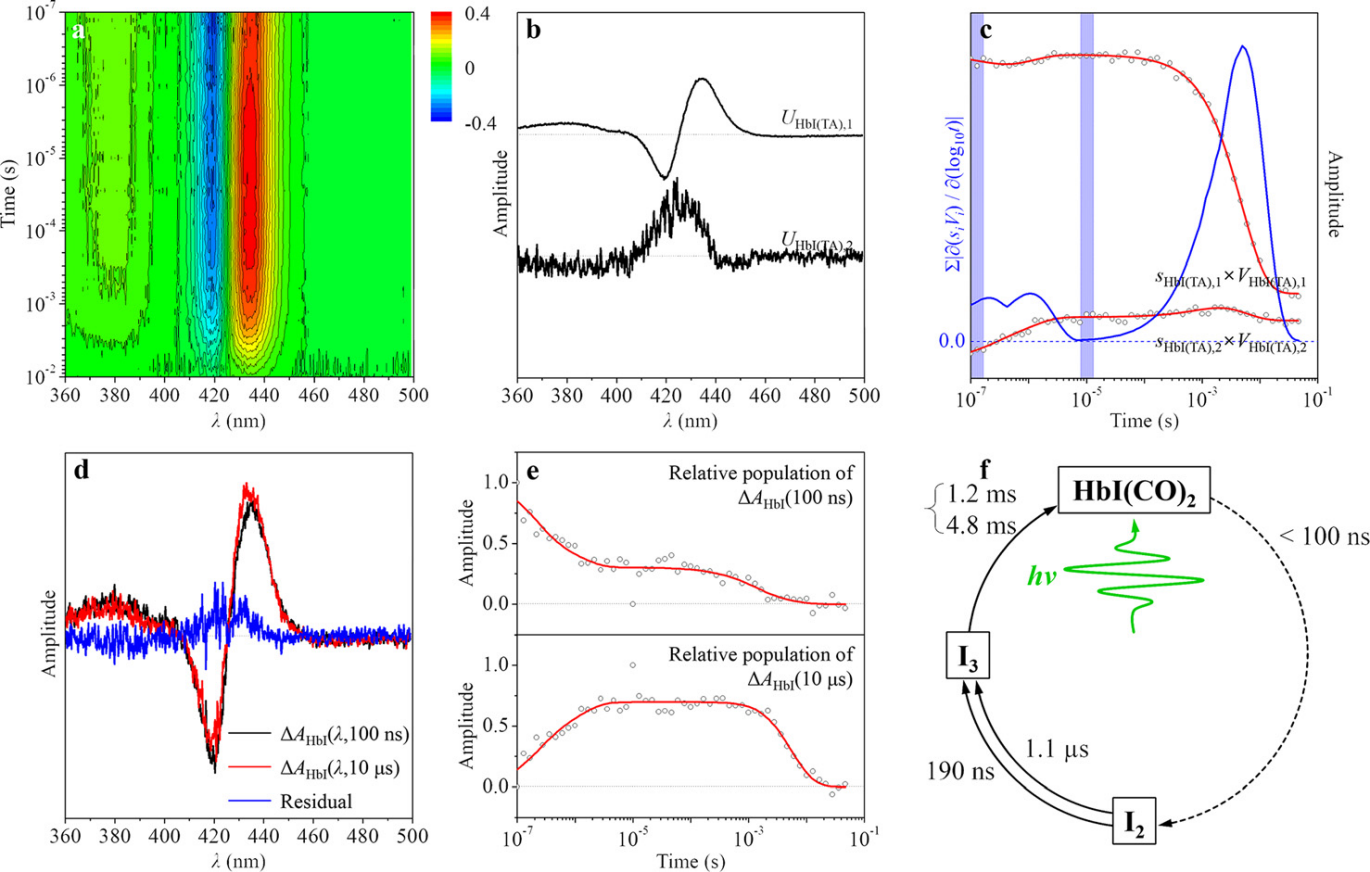
Example of the P method applied to TA data of wild-type HbI(CO)_2_. (a) Transient absorption spectra, Δ*A*_HbI_(*λ*,*t*), measured for a solution sample of wild-type HbI(CO)_2_. (b) Two principal time-independent lSVs. (c) Two principal time-dependent rSVs weighted by singular values (black circles). These time-dependent singular components were fit (red curves) by four exponentials sharing common relaxation times, yielding the relaxation times of 190 ± 100 ns, 1.1 ± 0.3 *μ*s, 1.2 ± 0.4 ms, and 4.8 ± 0.2 ms. Sum of the absolute values of the first derivatives of the principal rSVs weighted by singular values with respect to log_10_(time) (blue curve) was used as the selection criteria of the stationary time zones. We took the experimental curves within each of the two time zones shaded in blue color as pseudo SACs for two intermediates of HbI(CO)_2_ as shown in (d). (d) Pseudo SACs for two intermediates of HbI(CO)_2_ corresponding to the experimental TA spectra at two selected time delays, 100 ns (black curve) and 10 *μ*s (red curve). Blue curve shows the difference between two pseudo SACs. (e) Time-dependent relative populations (black circles) of the corresponding pseudo SACs. These populations were fit (red curves) by the relaxation times (190 ns, 1.1 *μ*s, 1.2 ms, and 4.8 ms) obtained from the weighted principal rSVs as shown in (c). (f) A kinetic model that well fits the experimental TA spectra of HbI(CO)_2_. This model is the truncated form of the optimum model shown in Figure 4(f).

To show the wide applicability of SAPPA, we also applied the P method to analyze time-resolved spectra, Δ*A*_HbI_(*λ*,*t*), of photoexcited wild-type HbI solution measured by transient absorption (TA) spectroscopy (Figure 5(a)). From SVD of the TA spectra in the *λ* range of 360–500 nm and the entire time range (100 ns–46.4 ms), we identified two principal singular components (possibly I_2_ and I_3_ species considering the time range) and four relaxation times (possibly two unimolecular time constants and one bimolecular time constant approximated by a combination of two latest unimolecular time constants considering the time range and the results of previous studies on HbI(CO)_2_^69,70^) as shown in Figures 5(b) and 5(c). There are two significant singular vectors, but we were able to identify only one stationary time zone (10 *μ*s) based on the time-dependent rSVs (black circles (experimental) and red curves (fit) in Figure 5(c)) and the sum of the absolute values of the first derivatives of the principal rSVs weighted by singular values with respect to log_10_(time) (blue curve in Figure 5(c)). The lack of stationary time zones compared with the significant singular vectors is due to limited time resolution (100 ns) of our TA measurement. As a result, we selected the TA spectra measured at 100 ns and 10 *μ*s as the pseudo SACs corresponding to I_2_ and I_3_ intermediates. We note that the pseudo SAC of the I_2_ intermediate had to be selected from the TA data measured at much later time delay (in this case, 100 ns) than the case of the TRXSS data (17.7 ns). Accordingly, the pseudo SAC of the I_2_ intermediate, Δ*A*_HbI_(*λ*,100 ns), shown in Figure 5(d) can be regarded as a mixture of pseudo SACs of I_2_ and I_3_ intermediates.

As shown in Figure 5(e), we found that the relative population of Δ*A*_HbI_(*λ*,100 ns) decays biphasically with time constants of 190 ns and 1.1 *μ*s and then further decays nonexponentially, which can be approximated by two dummy time constants of 1.2 ms and 4.8 ms. Subsequently, the population of Δ*A*_HbI_(*λ*,10 *μ*s) rises biphasically with time constants of 190 ns and 1.1 *μ*s and then decays nonexponentially, which can be approximated by two dummy time constants of 1.2 ms and 4.8 ms.^69,70^ By considering that Δ*A*_HbI_(*λ*,100 ns) is the mixture of the pseudo SACs of I_2_ and I_3_, the results allow us to determine the optimum kinetic model that involves (1) biphasic transition from the second intermediate to the third one due to the existence of two conformational substates of the second intermediate and (2) bimolecular nongeminate CO recombination of the third intermediate as shown in Figure 5(f). The relaxation times (190 ns and 1.1 *μ*s) determined by TA spectroscopy shown in Figure 5(f) are faster than those (730 ns and 5.6 *μ*s) probed by TRXSS (see Figure 4(f)), indicating that global structural changes of HbI may occur more slowly than local structural changes around the heme chromophore.^64,73^ This discrepancy in the time scales of transitions may be explained by considering the time taken for light-triggered local structural perturbation around a chromophore to propagate over the entire protein. Our recent study on E46Q mutant of PYP using both TRXSS and TA spectroscopy^64^ also reported that the global conformational change, which is observed by TRXSS, involved in the transition to the signaling state of the protein is temporally delayed from the local structural change around the chromophore, which is observed by TA spectroscopy. Thus, to have a complete understanding of protein structural dynamics, it is desirable to apply both TRXSS (sensitive to global structural changes of protein) and TA spectroscopy (sensitive to local structural changes of chromophore) or other optical spectroscopic techniques.^64^

## IV. CONCLUSION

In this work, we demonstrated the applicability of the SVD-aided pseudo principal-component analysis by examining the experimental TRXSS data of wild-type sperm whale MbCO and wild-type HbI(CO)_2_. In addition, we showed that SAPPA can be applied to time-resolved spectroscopic data as well by examining the experimental TA data of wild-type HbI(CO)_2_. This new method can be potentially used to easily determine the optimum kinetic model for various time-resolved data with high fidelity.

## V. SUPPLEMENTARY MATERIAL

See supplementary material for information on the TA experiment, the selection criteria of the stationary time zones, the result of a simulation where an intermediate transforms to another intermediate with two relaxation times, and the results of simulations where an intermediate, which has two conformational substates, transforms to another intermediate with two relaxation times.
